# Tellurite enters *Escherichia coli* mainly through the PitA phosphate transporter

**DOI:** 10.1002/mbo3.26

**Published:** 2012-06-19

**Authors:** Alex O Elías, María José Abarca, Rebecca A Montes, Thomas G Chasteen, José M Pérez-Donoso, Claudio C Vásquez

**Affiliations:** 1 Laboratorio de Microbiología Molecular, Departamento de Biología, Facultad de Química y Biología, Universidad de Santiago de ChileChile; 2 Escuela de Bioquímica, Universidad Andres BelloChile; 3 Department of Chemistry, Sam Houston State UniversityHuntsville, Texas

**Keywords:** *Escherichia coli*, phosphate transport, PitA, tellurite uptake

## Abstract

Several transporters suspected to be involved in tellurite uptake in *Escherichia coli* were analyzed. Results showed that the PitA phosphate transporter was related to tellurite uptake. *Escherichia coli* Δ*pitA* was approximately four-fold more tolerant to tellurite, and cell viability remained almost unchanged during prolonged exposure to the toxicant as compared with wild type or Δ*pitB* cells. Notably, reduced thiols (toxicant targets) as well as superoxide dismutase, catalase, and fumarase C activities did not change when exposing the Δ*pitA* strain to tellurite, suggesting that tellurite-triggered oxidative damage is attenuated in the absence of PitA. After toxicant exposure, remaining extracellular tellurite was higher in *E. coli* Δ*pitA* than in control cells. Whereas inductively coupled plasma atomic emission spectrometric studies confirmed that *E. coli* Δ*pitA* accumulates ∼50% less tellurite than the other strains under study, tellurite strongly inhibited ^32^P_i_ uptake suggesting that the PitA transporter is one of the main responsible for tellurite uptake in this bacterium.

## Introduction

Tellurite (TeO_3_^2−^), the most soluble tellurium oxyanion, is harmful to most organisms. Especially sensitive are Gram negative bacteria. In fact, early in the 20th century the antibacterial properties of penicillin and potassium tellurite were communicated (Fleming 1932). Nevertheless, high levels of tellurite resistance (Tel^R^) have been observed in different bacterial strains and at least five Tel^R^ determinants have been identified (Taylor 1999). Tel^R^ has been also related to *ter* genes (*terZABCDEF*). One or more *ter* genes have been found both in Gram positive and Gram negative bacteria (Taylor 1999). However, the mechanism by which *ter* genes allow tellurite resistance is still poorly understood and no specific function attributable to the proteins encoded by the *ter* gene cluster has been identified so far.

Currently, it is speculated that bacterial tellurite resistance is actually not related to specific gene products, but rather represents a multifactor response that is directly or indirectly involved in different metabolic pathways, their substrates and/or products (Avazéri et al. 1997; Turner et al. 1999, 2001; Vásquez et al. 2001; Pérez et al. 2007, 2008; Chasteen et al. 2009; Tremaroli et al. 2009).

In the recent years, available evidence suggests that at least part of TeO_3_^2−^ toxicity is due to the intracellular generation of reactive oxygen species (ROS) that occur concomitantly with tellurite (Te^4+^) reduction in its less toxic, elemental form (Te^0^) (Pérez et al. 2007; Tremaroli et al. 2007). Borsetti et al. (2005) reported that superoxide dismutase (SOD) activity increased as a consequence of exposing *Rhodobacter capsulatus* to TeO_3_^2−^, and that incubation with the ROS elicitor paraquat (Bus and Gibson 1984) resulted in increased cellular resistance to tellurite. In support of this, our group has shown that superoxide (O_2_^−^) is effectively generated during Te^+4^ to Te^0^ reduction (Pérez et al. 2007).

As TeO_3_^2−^ exerts its toxic effects once inside the cell, to get a global picture of the tellurite resistance/toxicity phenomena it is imperative to understand the mechanism(s) underlying the uptake of this toxicant. To date, there is no consensus about TeO_3_^2−^ entrance into cells. The first indication with regard to TeO_3_^2−^ uptake came from the work of Tomás and Kay (1986), who proposed that this toxicant would enter the cell through a phosphate transport system. Later, it was reported that in *R. capsulatus*, tellurite uptake is pH-dependent and completely inhibited by carbonyl cyanide *p*-trifluoromethoxy-phenylhydrazone (FCCP) and the K^+^/H^+^ exchanger nigericin (Borsetti et al. 2003). More recently, Borghese et al. (2008) reported that, in *R. capsulatus*, intracellular tellurite accumulation was inhibited by monocarboxylates as pyruvate, lactate, or acetate suggesting that TeO_3_^2−^ would enter this bacterium through a yet unidentified monocarboxylate transporter.

In this work, experiments with various strains lacking defined transporters were carried out to analyze their putative involvement in TeO_3_^2−^ uptake by *E. coli*. Initial results showed that, as suggested earlier by Tomás and Kay (1986), cells defective in phosphate transport were less sensitive to this toxicant, suggesting a possible role of this system in TeO_3_^2−^ uptake. These strains were selected for further analysis and results showed that one of the main routes of TeO_3_^2−^ entrance into *E. coli* is the PitA symporter.

## Experimental Procedures

### Bacterial strains and growth conditions

*Escherichia coli* BW25113 (lacI^q^ *rrnB*_T14_ ΔlacZ_WJ16_ hsdR514 Δ*araBAD*_AH33_ Δ*rhaBAD*_LD78_, wild type) and its isogenic mutant derivatives JW3460 (*pitA::kan*) and JW2955 (*pitB::kan*) were from the KEIO collection of the National Institute of Genetics, Japan (Baba et al. 2006). *Escherichia coli* AG1 (*endA*1 *recA*1 *gyrA*96 *thi*-1 *relA*1 *glnV*44 *hsdR*17 [r_K_^−^ m_K_^+^], wild type) and its ASKA derivatives (Kitagawa et al. 2005) that over-express the cloned genes in the presence of IPTG were obtained from the same source. Plasmid pCN24A containing the cloned *E. coli pitA* gene (p*pitA*) was purified from the corresponding PitA^ASKA^ strain (NARA Institute, Japan) using the Qiagen (Germany) plasmid purification kit and transformed into *E. coli* JW3460.

Cells were routinely grown in M9 minimal medium (Sambrook et al. 1989) supplemented with 0.2% glucose. When required, kanamycin (10 μg mL^−1^) was added. *Escherichia coli* AG1 and derived strains were grown in LB medium supplemented with chloramphenicol (30 μg mL^−1^). Unless otherwise indicated, tellurite concentration in the different assays was 20 μmol/L.

### Determination of growth inhibition areas and minimal inhibitory concentration (MIC)

Bacteria were evenly spread in LB agar (2%) plates or M9 minimal medium plates (containing 0.2% glucose) amended with the appropriate antibiotics. Growth inhibition zones (GIZs) were determined as described earlier (Fuentes et al. 2007).

Tellurite MIC was assessed in M9 liquid medium supplemented with 0.2% glucose. Aliquots (5 μL) of overnight cultures were mixed in a 96-well microplate with 200 μL of medium containing increasing TeO_3_^2−^ concentrations. Serial dilutions were performed starting with a sterile 400 μmol/L TeO_3_^2−^ solution. After 12 h at 37°C, the minimal toxicant concentration inhibiting bacterial growth was determined.

### Cell viability assays

Saturated cultures of *E. coli* BW25113 or its mutant derivatives were diluted (1:100) with fresh M9-glucose medium and incubated at 37°C with constant shaking to OD_600_ ∼0.05. The culture was again diluted 1:100 with the same medium prewarmed to 37°C and growth was continued to OD_600_ ∼0.15. Then, TeO_3_^2−^ was added (controls received sterile water) and at different time intervals, aliquots were taken, diluted 10^6^-fold, and plated in M9-glucose. After incubating overnight at 37°C, the number of colony forming units (CFU) was determined.

### Determination of thiol concentration

Overnight cultures were diluted 1:100 with fresh M9-glucose medium and shaked at 37°C until OD_600_ ∼0.15. Tellurite was added and incubation was continued for an additional 15 min. Controls received no toxicant. The amount of total thiols (RSH) was determined as described by Turner et al. (1999) using a molar extinction coefficient of 1.36 × 10^−4^ M^−1^ cm^−1^ (oxidized DTNB) (Riddles et al. 1983).

### Preparation of protein extracts

Cell cultures were sedimented by centrifugation at 4°C, washed 2× with 2 mL of 50 mmol/L potassium phosphate pH 7.4 buffer, and suspended in 1 mL of the same buffer. After adding the protease inhibitor PMSF (1 mmol/L), cells were disrupted by sonication. The cell debris was discarded by centrifugation at 12,000 *g* for 10 min at 4°C. The supernatant was considered as the crude extract. Protein concentration was determined as described (Bradford 1976).

### Determination of enzyme activity

#### Superoxide dismutase

SOD was assayed in the crude extract (30 μg protein) using the xanthine-xanthine oxidase system as reported previously (McCord and Fridovich 1969).

#### Catalase

The enzyme was assayed for 2 min by monitoring H_2_O_2_ decomposition at 240 nm. The reaction mix (1 mL) contained 50 mmol/L potassium phosphate pH 7.0 buffer and 19.4 mmol/L H_2_O_2_. The reaction was started with the crude extract (45 μg protein) as described (Chen and Schellhorn 2003).

#### Fumarase C

Fumarase C activity was assessed in the crude extract (15 μg protein) by measuring the formation of fumarate from l-malate for 2 min at 250 nm. A molar extinction coefficient of 1.62 mM^−1^ cm^−1^ was used (Liochev and Fridovich 1992).

#### Extracellular tellurite concentration

*Escherichia coli* grown to OD_600_ ∼0.15 was treated with 20 μmol/L tellurite and aliquots were taken at various time intervals to determine remaining tellurite in the supernatant as described previously (Molina et al. 2010).

#### Inductively coupled plasma atomic emission spectrometry

Intracellular tellurite concentration was determined by inductively coupled plasma atomic emission spectrometry (ICP-AES). Briefly, cells were grown to OD_600_ ∼0.7 and after adding tellurite (80 μmol/L) the culture was incubated for additional 15 min. After centrifuging at 10,000 *g* for 5 min, the pellet was washed twice with sterile Millipure water, heated and taken to dryness with concentrated HNO_3_, and then redissolved in 10 mL of 10% nitric acid. Samples were analyzed using a Spectro CIROS Vision ICP-AES instrument. The Te analytical line was 214.281 nm.

#### Preparation of right-side-out membrane vesicles (RSOVs)

RSOVs were obtained as described by Kaback (1971). The final pellet was suspended in 50 mmol/L Tris-HCl pH 6.6 buffer (4–7 mg mL^−1^ protein) and used immediately.

#### ^32^P_i_ uptake studies

Quantification of radioactive inorganic phosphate (^32^P_i_) uptake using whole cells was performed according to the method of Poole and Hancock (1984) with some modifications. Briefly, cells were grown overnight in “Phosphate-Free Buffered Media” (PFBM; 50 mmol/L triethanolamine, 15 mmol/L KCl, 10 mmol/L [NH_4_]_2_SO_4_, and 1 mmol/L MgSO_4_) and centrifuged at 8500 *g* for 3 min. After washing 3× with the same buffer, cells were suspended to an OD_600_ ∼0.25 with 50 mmol/L Tris-HCl pH 6.9 buffer that contained 10 mmol/L MgSO_4_ and kept on ice until use. The suspension was warmed to 37°C for ∼10 min and aliquots (1 mL) were incubated with ^32^P_i_ with constant stirring. Two hundred microliter aliquots were removed at different time intervals and, after a brief centrifugation, the supernatant was directly loaded onto glass-fiber filters. The pellet was washed twice with 50 mmol/L Tris-HCl pH 6.9 buffer, suspended with 200 μL of the same buffer, and deposited on clean filters. Filters were allowed to dry for 12 h and radioactivity was determined by liquid scintillation counting. Controls included 200 μL of buffer with no ^32^P_i_ (background counts) or ^32^P_i_ without cells (maximal counts). Quantification of ^32^P_i_ uptake by RSOVs prepared from *E. coli* AG1 or PitA^ASKA^ was carried out according to Seol and Shatkin (1992).

#### Tellurite-phosphate competition assays

Cell suspensions (200 μL) obtained as stated above were incubated with ^32^P_i_ in the presence of increasing TeO_3_^2−^ concentrations for 8 min with constant agitation at room temperature. After a brief centrifugation, the supernatant was discarded and the pellet washed twice and suspended in 100 μL of 50 mmol/L Tris-HCl pH 6.9 buffer to determine radioactivity. Competition assays using RSOVs were carried out as described for whole cells.

### Statistical analysis

Statistical analysis of data was carried out using the GraphPad Prism® 5 software. Analysis of variance (ANOVA) was at *P* <0.05.

## Results

As mentioned above, tellurite becomes toxic only once inside the cell. In this context, we and others have been interested in trying to unveil the toxicant's entry pathway. To date, there is still some controversy about it, and different systems involved in transporting important biological molecules have been related to the process.

Initially, a number of *E. coli* strains lacking defined transport systems were tested for tellurite tolerance using classical microbiological procedures as the determination of GIZs and MIC. The rationale was that if a particular carrier was actually involved in tellurite uptake, cells lacking it should exhibit increased tolerance to the toxicant. Several strains from the KEIO knockout collection (Baba et al. 2006) lacking defined transport systems were screened for tellurite resistance. These included mutants lacking (i) PitA or PitB inorganic phosphate (Pi) transport systems, (ii) acetate (ActP) or lactate (LacY) carriers (Borghese and Zannoni 2010), (iii) YghK glycolate transporter (Núñez et al. 2001), (iv) PanF pantothenate transporter (Hosie et al. 2002), and (v) the hypothetical transporter TsgA (Guzzo and Dubow 2000) (Table S1). As preliminary observations from our laboratory showed that *E. coli* grown in the presence of arabinose became more sensitive to tellurite, in addition, AraE and AraJ transporters were considered. Finally, to examine the possible involvement of ATP-dependent, highly specific arabinose or Pi transport, mutants lacking AraF or AraG from the AraFGH operon, the phosphate-binding periplasmic protein PstS, or the catalytic subunit of the PstSCAB complex PstB (Torriani 1990) were also analyzed.

Growth inhibition areas and tellurite MICs for these various strains clearly showed that cells lacking the component of the Pit phosphate transport system and PitA, were more tolerant to tellurite as compared with the control strain. In a control experiment, PitA-lacking cells were transformed with the cloned *E. coli pitA* gene. As expected, Δ*pitA/*p*pitA* cells displayed a similar phenotype to that observed for wild type and Δ*pitB* cells, as evidenced by MIC and GIZ determinations (Table 1).

**Table 1:** Minimal inhibitory concentration (MIC, μmol/L) of tellurite and growth inhibition zones (GIZ, cm^2^) for the indicated *Escherichia coli* strains

	MIC		GIZ	
		
*E. coli*	LB	M9	LB	M9
BW25113	2.5	50	7.9 ± 0.3	7.0 ± 1.0
Δ*pitA*	12.0	200	6.6 ± 0.5	4.8 ± 0.7
Δ*pitA/*p*pitA*	3.0	50	8.1 ± 0.3	7.3 ± 0.1
Δ*pitB*	4.0	80	6.8 ± 0.3	6.2 ± 0.5

Assays were carried out in LB or M9 minimal culture media as described in Experimental Procedures (*n* = 5).

To minimize the effects of a complex growth medium, but with higher phosphate concentrations, glucose-amended M9 minimal medium (Sambrook et al. 1989) was used in all the following experiments. The Δ*pitA* strain showed the highest tellurite MIC and GIZ, suggesting the involvement of this transporter in tellurite uptake by *E. coli*. When cell viability of tellurite-exposed cultures was analyzed, all strains exhibited a significant decrease in the number of viable cells, excepting the PitA-lacking mutant (Fig. S1).

**Figure 1:**
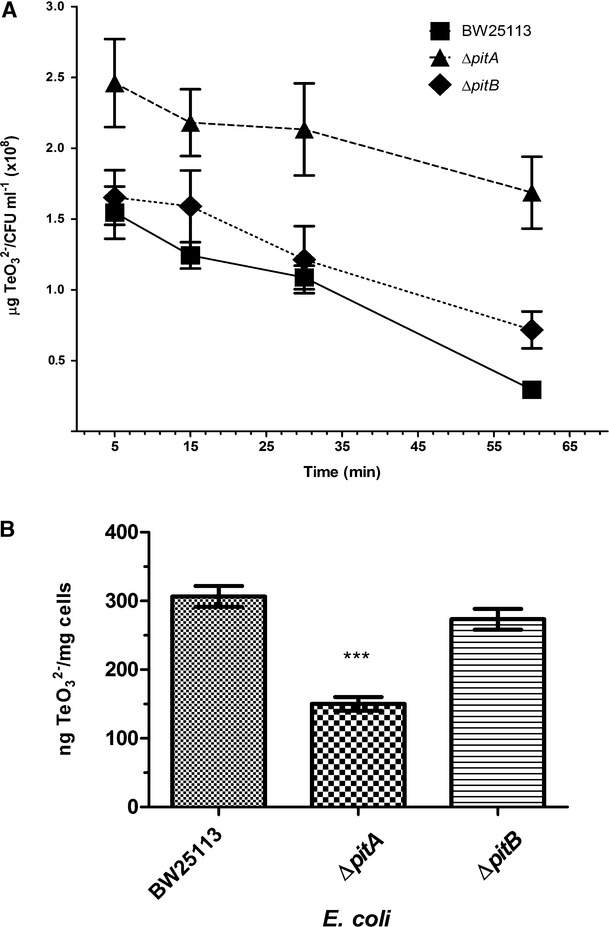
(A) Remaining extracellular tellurite in the indicated *Escherichia coli* strains. Cells were grown to OD_600_ ∼0.15 in M9-glucose medium, exposed to 20 μmol/L TeO_3_^2−^, and the remaining tellurite was determined in the supernatant at the indicated times. Results were normalized to the number of colony-forming units (CFU) mL^−1^. Bars represent the standard deviation (*n* = 9). (B) Intracellular tellurite content. Cells were grown to OD_600_ ∼0.8, exposed to TeO_3_^2−^ and growth was continued for an additional 15 min. Accumulated intracellular tellurite was determined by ICP-AES as described in Experimental Procedures. Results were normalized by the cell mass (mg of cells, wet weight). Bars represent the standard deviation (*n* = 3).

Next, tellurite uptake was analyzed. As in previous experiments, cells were treated with the toxicant for different time intervals (5, 15, 30, and 60 min), and extracellular remaining tellurite was determined as described by Molina et al. (2010). Figure 1A shows that after 30 min, tellurite remained almost unchanged in the supernatant of the Δ*pitA* strain, in contrast to the situation with wild type and Δ*pitB* strains, strongly suggesting that toxicant uptake is mediated by PitA. Almost identical results were obtained with spheroplasts prepared from all the tested strains, ruling out an effect of the *E. coli* outer membrane in toxicant's bioavailability. It was also found that, as occurs with phosphate, tellurite entrance also depends on the presence of a divalent ion (Mg^2+^ or Mn^2+^, not shown). Intracellular tellurite accumulated by wild type and PitA- or PitB-lacking cells was determined by ICP-AES. As expected, it was found that Δ*pitA* cells accumulated ∼50% less toxicant than the other two strains (Fig. 1B). These results clearly indicate that the absence of PitA largely prevents the entry of the toxicant into the cell, thus supporting the previous findings.

**Figure 2:**
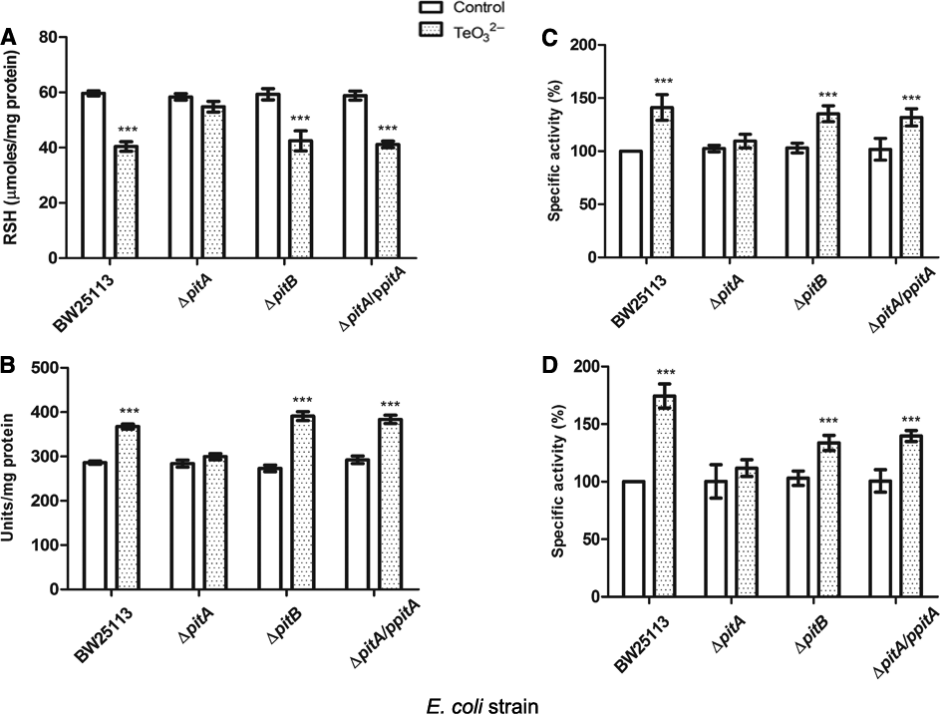
(A) Total RSH in the indicated *Escherichia coli* strains. Thiols were determined in extracts of tellurite-exposed *E. coli* using the DTNB reagent as described in Experimental Procedures. (B) SOD activity in wild type and the indicated mutant *E. coli* strains. Saturated cultures were diluted 1:100 with fresh M9-glucose minimal medium and grown to OD_600_ ∼0.15. Then 20 μmol/L TeO_3_^2−^ was added, and after 30 min cells were harvested and disrupted by sonication. SOD activity was determined as described in Experimental Procedures. (C and D) Specific catalase (C) and FumC (D) activity in *E. coli* exposed to TeO_3_^2−^. Toxicant exposure was 30 and 15 min, respectively. Enzyme activity was determined as described in Experimental Procedures. Bars represent the standard deviation (*n* = 6).

If these observations are true, then Δ*pitA* cells should exhibit lower tellurite-induced damage to macromolecules. One of the main detrimental tellurite effects is the depletion of reduced cellular thiols (RSH), glutathione being the main target (Turner et al. 2001). In this context, RSH levels were determined in wild type *E. coli* as well as in the Pit mutants. Figure 2A shows the levels of reduced thiols in the control condition (without amendment) and in cells exposed to tellurite (20 μmol/L) for 15 min. As expected, RSH levels did not change in Δ*pitA* cells after tellurite exposure, whereas those observed in wild type and Δ*pitB* strains decreased ∼40%, supporting the idea of a relationship between the absence of this transporter and increased tolerance to the toxic.

On the other hand and as tellurite-exposed cells show increased SOD activity (Pérez et al. 2007), it was of interest to assess SOD activity in the strains under study, exposed or not to the toxicant. Figure 2B shows that whereas SOD activity increased both in wild type and Δ*pitB* strains, it remained virtually unchanged in Δ*pitA* cells. In the same context, the activity of tellurite-sensitive enzymes that respond later in the detoxification process was examined. Catalase activity increased ∼40% in wild type and Δ*pitB* strains when exposed to tellurite. Conversely, no changes in enzyme activity were observed in Δ*pitA* cells (Fig. 2C). Similar results were observed when assaying FumC (fumarase C). FumC activity increased 40–75% in all strains except Δ*pitA* (Fig. 2D), suggesting that cells lacking PitA undergo less tellurite-induced oxidative damage because of a lower intracellular tellurite concentration, or a diminished toxicant entrance, or both. As expected, genetically complemented Δ*pitA* displayed a similar phenotype to that of wild type and Δ*pitB* cells.

**Figure 3:**
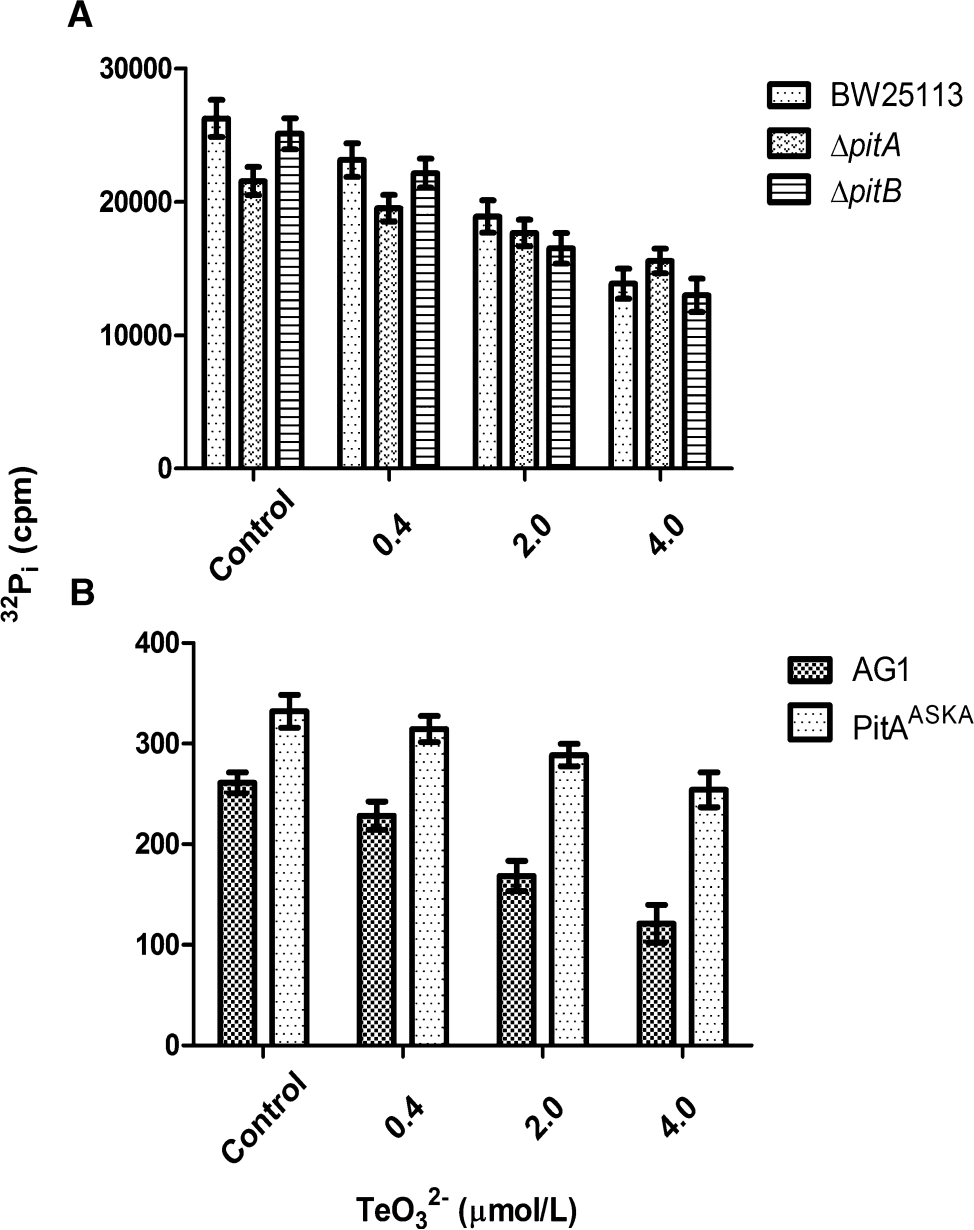
(A) Tellurite effect on ^32^P_i_ uptake by *Escherichia coli*. Cells were assayed for phosphate uptake (8 min) in the presence of the indicated tellurite concentrations. Assays were performed as described in Experimental Procedures. Control contained no tellurite. (B) Tellurite effect on ^32^P_i_ uptake by RSOVs generated from the indicated bacterial strains. Tests were conducted as with whole cells. Controls contained no tellurite. Bars represent the standard deviation (*n* = 3).

Finally, ^32^P_i_ transport was analyzed in wild type and the Δ*pitA* and Δ*pitB* strains. Extra and intracellular ^32^P_i_ levels indicated a similar behavior for wild type and Δ*pitB E. coli*: a decrease in the isotope in the supernatant was paralleled by an intracellular increase in ^32^P_i_ (Fig. S2A and C). Unlike these strains, Δ*pitA* cells showed a less pronounced isotope uptake (Fig. S2B), consistent with PitA being responsible for at least 90% of P_i_ transport in normal growth conditions (Harris et al. 2001). Increasing tellurite concentration caused a decrease in the amount of ^32^P_i_ transported into the cell in all strains tested. While intracellular isotope levels were decreased by ∼60% in wild type and Δ*pitB E. coli*, the Δ*pitA* strain showed an inhibition of isotope incorporation not exceeding ∼10% of that observed in control cells (Fig. 3A). As expected, isotope uptake by PitA-enriched RSOVs also increased significantly as compared with the isogenic wild type strain AG1 (Fig. S3). Tellurite also inhibited P_i_ uptake by AG1 vesicles, reaching ∼45% inhibition in the presence of 2 μmol/L tellurite (Fig. 3B). In the case of PitA-enriched vesicles, a light (∼10%) inhibitory effect was observed only in the presence of the maximum tellurite concentration tested (4 μmol/L). When higher toxicant concentrations were used, the intracellular ^32^P_i_ decreased ∼60% (not shown).

## Discussion

In 1986, Tomás and Kay proposed that tellurite uptake would involve the participation of the phosphate transport system (Tomás and Kay 1986). Although this was the first time that a specific transport system was associated with tellurite uptake, some features of this phenomenon had already been studied by Cooper and Few (1952), who postulated that tellurite transport is a fast phenomenon that occurs mainly within the first minutes of exposure.

The proposal of Tomás and Kay was generally accepted until Borsetti et al. (2003) showed that TeO_3_^2−^ uptake was pH-dependent and completely inhibited by FCCP and nigericin in *R. capsulatus*. The same group proposed that a not-yet-characterized monocarboxylate transporter was responsible for toxicant uptake in this rod (Borghese et al. 2008). More recently, Borghese et al. proposed that the main transporter system responsible for TeO_3_^2−^ uptake in *R. capsulatus* is the acetate carrier ActP (Borghese and Zannoni 2010). Later, it was demonstrated that fructose abolishes *actP* gene expression causing a sharp decrease of TeO_3_^2−^ uptake, which resulted in increased tolerance of *R. capsulatus* to potassium tellurite (Borghese et al. 2011). Notwithstanding these results, ActP actually does not exhibit the characteristics of a tellurite transporter, that is, it is neither pH-dependent nor completely inhibited by FCCP.

An ideal tellurite transporter would be a proton-symport system whose substrate would share some structural and/or functional connection with tellurite. In this regard, the observation of Tomás and Kay (1986) is supported as there is a phosphate transport system involved that meets precisely these requirements. The proton-symport system PitAB is completely inhibited by FCCP and can also transport arsenate nonspecifically (Veen 1997). Although phosphate transport is inhibited significantly by both metalloids, tellurite is at least 200-fold more potent than arsenate (Tomás and Kay 1986).

Figure 1A shows that remaining extracellular tellurite levels decrease with incubation time. Whereas both wild type and Δ*pitB* cells transport tellurite actively, Δ*pitA* cells maintain toxicant uptake relatively constant up to 30 min, which is consistent with the unchanged levels of total RSH and enzyme activities observed in these cells upon tellurite exposure (Fig. 2). Incorporated tellurite was then assessed by ICP-AES. Figure 1B clearly shows that toxicant levels accumulated by BW25113 and Δ*pitB* cells are very similar. In contrast, intracellular tellurite in the Δ*pitA* strain decreased by ∼50%.

As mentioned above, various indicators of intracellular damage by tellurite exposure were assessed. For instance, lowering tellurite uptake by means of deleting a particular transporter should be reflected in RSH levels changes. This was precisely what happened in wild type and Δ*pitB E. coli*, where the RSH pool dropped ∼50% after 15 min of exposure to the toxicant. Under the same conditions, the absence of PitA resulted in undetectable disturbances in total RSH content (Fig. 2A). Furthermore, SOD activity increased ∼40% in all strains under toxicant exposure, except for Δ*pitA* cells (Fig. 2B), reinforcing the idea that the absence of PitA decreases effectively the intracellular tellurite, thus lowering superoxide levels and hence *sodA* induction. On the other hand and as expected, the activity of enzymes that respond later during the process of tellurite detoxification (CAT and FumC) increased in all strains, but Δ*pitA* under tellurite-exposure (Fig. 2C and D).

According to these observations, phosphate uptake should be significantly altered in the presence of tellurite. In fact, whereas the uptake of this molecule was rather linear with time in BW25113 and Δ*pitB* cells, ^32^P_i_ incorporation by Δ*pitA* cells was almost constant (Fig. S2).

When assaying the effect of tellurite on isotope uptake, an inverse relationship between the amount of tellurite present in the assay and ^32^P_i_ uptake was observed in all strains tested. The exception was the Δ*pitA* strain, for which isotope levels did not depend on tellurite concentrations and remained almost constant (Fig. 3A). This observation supports the idea that PitA is one of the primary transporter responsible for tellurite uptake in *E. coli*, its absence resulting in decreased phosphate transport. Tellurite seems to slightly alter phosphate uptake in PitA-lacking cells, presumably through PitB. As there is a great nucleotide sequence identity between *pitA* and *pitB*, one would expect a similar ability to transport the toxicant. However, to function as effectively as PitA, PitB would require forming a multicopy complex (Harris et al. 2001).

Over expressing PitB results in increased intracellular tellurite, as determined by ICP-AES (not shown). However, its absence does not seem to cause significant impact in terms of toxicant tolerance and/or phosphate uptake. Whereas the constitutively expressed PitA is responsible for ∼95% of P_i_ transport, PitB seems to be regulated by the amount of available phosphate and would be under the control of the *pho* regulon (Harris et al. 2001).

Finally, uptake as well as tellurite-inhibited phosphate transport by PitA-enriched RSOVs was assessed. Results showed a direct relationship between PitA amount and intravesicle ^32^P_i_ (Fig. S3A and B). Similarly to that observed with whole cells, an inverse relationship between the amount of isotope accumulated and tellurite concentration was observed. PitA-enriched RSOVs showed higher levels of intra-vesicle ^32^P_i_, but the inhibitory tellurite effect on phosphate uptake was not observed. Actually, inhibition did not exceed 10%, which could be explained by the large amount of PitA in the vesicles. Thus, tested tellurite concentrations were not enough to prevent normal phosphate transport. Higher concentrations of toxicant (over 8.0 μmol/L) resulted in decreased phosphate accumulation (not shown).

Summarizing, results from this work strongly suggest that an important route of tellurite entrance into *E. coli* is represented by the PitA phosphate transporter. The Pit system requires necessarily the presence of a divalent cation (Me = Mg^2+^, Ca^2+^) to form a soluble, neutral metal-phosphate complex (MeHPO_4_), which is the species that is symported along with a proton (van Veen 1997). Under normal growth conditions, one might expect that the periplasm's pH be similar to that of the culture medium (normally 7.0–7.4). Given the pKa values for H_3_PO_4_ dissociation (pKa_1_ 2.19 and pKa_2_ 6.94), the predominant species H_2_PO_4_^−^ and HPO_4_^2−^ could form the complex in the presence of a Me^2+^ excess. At the same pH, tellurite is present as TeO_3_^2−^ and HTeO_3_^−^ which could form a complex with similar characteristics that could be transported – via PitA – into the cell. Additional experiments to clarify this issue are under way at our laboratory.

## Supporting Information

**Table S1.** *Escherichia coli* strains used in this study.

**Figure S1.** Cell viability of tellurite-exposed *Escherichia coli*. Cell viability was determined as described in Experimental Procedures. Growth curves for each strain in the absence of toxicant were almost identical, so this situation is represented by the control curve. Bars represent the standard deviation (*n* = 12).

**Figure S2.** ^32^P_i_ uptake by *Escherichia coli* BW25113 (A), Δ*pitA* (B), and Δ*pitB* cells (C). Assays were carried out as described in Experimental Procedures.

**Figure S3.** ^32^P_i_ uptake by membrane vesicles from strains AG1 (A) and PitA^ASKA^ (B). Assays were performed as described in Experimental Procedures using a RSOVs amount equivalent to a protein concentration of 40 μg mL^−1^. Bars represent the standard deviation (*n* = 3).
