# Supplementary material for: Tellurite enters Escherichia coli mainly through the PitA phosphate transporter
Source: Microbiologyopen. 2012 Jun 19;1(3):259–67. doi: 10.1002/mbo3.26 (PMC3501828; doi:10.1002/mbo3.26)
Supplement: Supplementary file 1 [file mbo30001-0259-SD1.doc]

**Table S1.** *E. coli* strains used in this study

| *E. coli* | Description | Relevant genotype | Source or reference |
| --- | --- | --- | --- |
| BW25113 | Wild type | F-, Δ(*araD*-*araB*)567, Δ*lac*Z4787(::*rrn*B-3), λ-, Δ(*rhaD*-*rhaB*)568, *hsdR*514 | KEIO Collection, Nara Institute, Japan |
| JW3460 | Pi transport | Δ*pitA::kan* |
| JW2955 | Pi transport | Δ*pitB::kan* |
| JW4028 | Acetate transporter | Δ*actP::kan* |
| JW3327 | Putative transporter | Δ*tsgA::kan* |
| JW2809 | Arabinose transporter | *ΔaraE::kan* |
| JW0386 | Putative arabinose transporter | *ΔaraJ::kan* |
| JW1889 | Arabinose periplasmic binding protein | *ΔaraF::kan* |
| JW1888 | ATP-binding component of the AraFGH system | *ΔaraG::kan* |
| JW3706 | Pi periplasmic binding protein | *ΔpstS::kan* |
| JW3703 | ATP-binding component of the PstSCAB system | *ΔpstB::kan* |
| JW3578 | Lactose transporter | *ΔlacY::kan* |
| JW3226 | Pantothenate transporter | *ΔpanF::kan* |
| JW2942 | Glycolate transporter | *ΔyghK::kan* |
