# Supplementary figures and images for: Tellurite enters Escherichia coli mainly through the PitA phosphate transporter
Source: Microbiologyopen. 2012 Jun 19;1(3):259–67. doi: 10.1002/mbo3.26 (PMC3501828; doi:10.1002/mbo3.26)

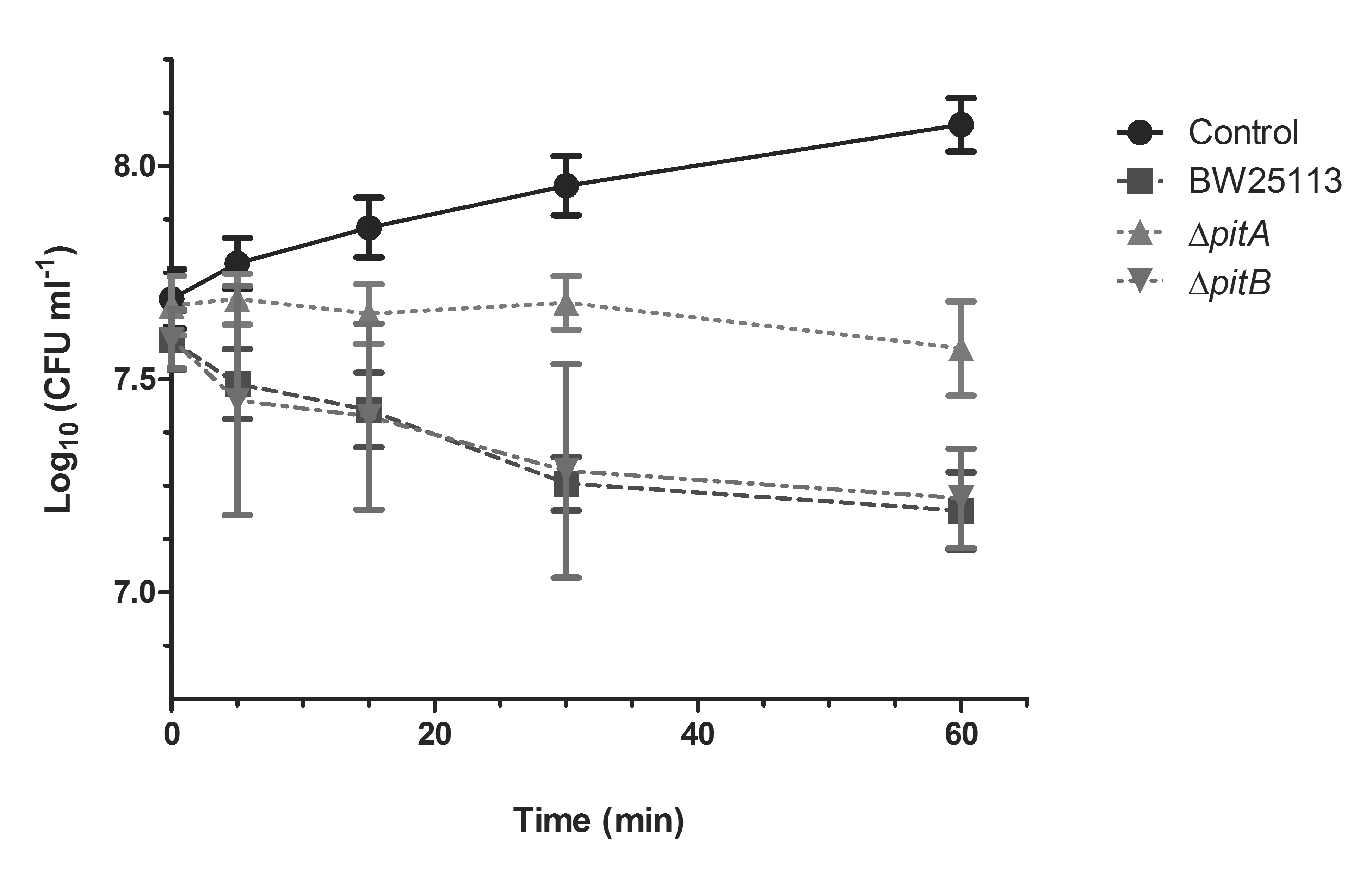

Supplement: Supplementary file 2 [file mbo30001-0259-SD1.tif]

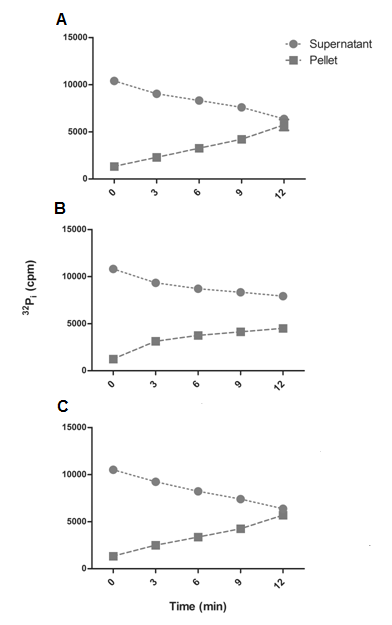

Supplement: Supplementary file 3 [file mbo30001-0259-SD2.tif]

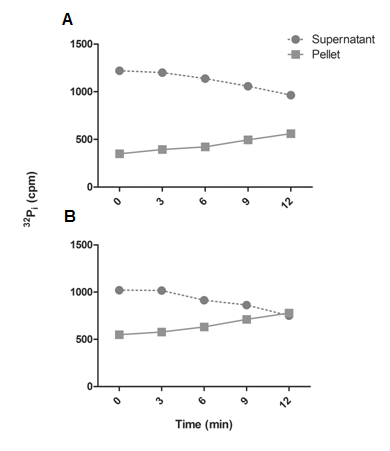

Supplement: Supplementary file 4 [file mbo30001-0259-SD3.tif]
